# KCa3.1 Mediates Dysregulation of Mitochondrial Quality Control in Diabetic Kidney Disease

**DOI:** 10.3389/fcell.2021.573814

**Published:** 2021-02-19

**Authors:** Chunling Huang, Hao Yi, Ying Shi, Qinghua Cao, Yin Shi, Delfine Cheng, Filip Braet, Xin-Ming Chen, Carol A. Pollock

**Affiliations:** ^1^Kolling Institute, Sydney Medical School Northern, Faculty of Medicine and Health, University of Sydney, Royal North Shore Hospital, Sydney, NSW, Australia; ^2^Division of Nephrology, School of Medicine, Stanford University, Stanford, CA, United States; ^3^Discipline of Anatomy and Histology, School of Medical Sciences, Faculty of Medicine and Health, The Bosch Institute, University of Sydney, Sydney, NSW, Australia; ^4^Australian Centre for Microscopy and Microanalysis, University of Sydney, Sydney, NSW, Australia

**Keywords:** diabetic kidney disease, mitochondrial quality control, mitochondrial dynamics, mitophagy, transforming growth factor β1, KCa3.1

## Abstract

Mitochondrial dysfunction is implicated in the pathogenesis of diabetic kidney disease. Mitochondrial quality control is primarily mediated by mitochondrial turnover and repair through mitochondrial fission/fusion and mitophagy. We have previously shown that blockade of the calcium-activated potassium channel KCa3.1 ameliorates diabetic renal fibrosis. However, the mechanistic link between KCa3.1 and mitochondrial quality control in diabetic kidney disease is not yet known. Transforming growth factor β1 (TGF-β1) plays a central role in diabetic kidney disease. Recent studies indicate an emerging role of TGF-β1 in the regulation of mitochondrial function. However, the molecular mechanism mediating mitochondrial quality control in response to TGF-β1 remains limited. In this study, mitochondrial function was assessed in TGF-β1-exposed renal proximal tubular epithelial cells (HK2 cells) transfected with scrambled siRNA or KCa3.1 siRNA. *In vivo*, diabetes was induced in KCa3.1+/+ and KCa3.1−/− mice by low-dose streptozotocin (STZ) injection. Mitochondrial fission/fusion-related proteins and mitophagy markers, as well as BCL2 interacting protein 3 (BNIP3) (a mitophagy regulator) were examined in HK2 cells and diabetic mice kidneys. The *in vitro* results showed that TGF-β1 significantly inhibited mitochondrial ATP production rate and increased mitochondrial ROS (mtROS) production when compared to control, which was normalized by KCa3.1 gene silencing. Increased fission and suppressed fusion were found in both TGF-β1-treated HK2 cells and diabetic mice, which were reversed by KCa3.1 deficiency. Furthermore, our results showed that mitophagy was inhibited in both *in vitro* and *in vivo* models of diabetic kidney disease. KCa3.1 deficiency restored abnormal mitophagy by inhibiting BNIP3 expression in TGF-β1-induced HK2 cells as well as in the diabetic mice. Collectively, these results indicate that KCa3.1 mediates the dysregulation of mitochondrial quality control in diabetic kidney disease.

## Introduction

Mitochondria are responsible for the main site of adenosine triphosphate (ATP) synthesis via oxidative phosphorylation (Alpers and Hudkins, 2011). Mitochondria have also been shown to play a crucial role in calcium signaling, reactive oxygen species (ROS) generation, apoptosis, necrosis, and innate immunity (Galluzzi et al., 2012; Suarez-Rivero et al., 2016). Mitochondrial dysfunction is characterized by a decrease in ATP production and increase in ROS generation leading to oxidative stress (Suarez-Rivero et al., 2016). Hence, maintaining optimal function of the mitochondria is important for maintaining cell survival, regulating cell death and cellular metabolic homeostasis (Sharma et al., 2003).

Mitochondrial quality control is exquisitely regulated to maintain functional mitochondria (Sharma et al., 2003). Mitochondrial quality control mechanisms are mainly regulated by mitochondrial dynamics and mitophagy (Ranjit et al., 2016). Mitochondrial dynamics include fission and fusion to repair or delete damaged components of the mitochondria. Mitochondrial fission allows for the segregation of damaged mitochondria, while mitochondrial fusion facilitates the exchanging of material between healthy mitochondria. Imbalanced mitochondrial fission and fusion are detrimental to mitochondrial function and cellular survival. Mitochondrial dynamics are regulated by several different GTPase proteins. Mitochondrial fission is regulated by dynamin-related protein 1 (Drp1) and mitochondrial fission protein 1 (Fis1). Mitochondrial fusion is mediated by mitofusin 1 (Mfn1), mitofusin 2 (Mfn2), and optic atrophy 1 (Opa1) proteins. Mfn1 and Mfn2 are localized on the mitochondrial outer membrane (MOM) and mediate tethering of MOM of adjacent mitochondria to promote the fusion of MOM, whereas Opa1 is responsible for mitochondrial inner membrane (MIM) fusion (Anand et al., 2014). Abnormalities in these mitochondrial dynamic proteins lead to severely altered mitochondrial morphology, defective mitochondrial function, and eventually cell death (Zhan et al., 2013). Mitophagy is selective autophagy to degrade and recycle dysfunctional or damaged mitochondria. Recent studies suggest that mitochondrial priming is mediated either through the Pink1/Parkin signaling pathway or the mitophagic receptors such as BCL2 interacting protein 3 (BNIP3), BNIP3 like (BNIP3L/NIX), and FUN14 domain containing 1 (FUNDC1) (Li et al., 2018; Wang J. et al., 2020). Disruption of mitochondrial networks prevents the elimination of damaged mitochondria and exacerbates ATP deficits, which is then implicated in a variety of diseases including diabetic kidney disease (Forbes and Thorburn, 2018; Suomalainen and Battersby, 2018). Although dysfunctional mitochondria are increasingly recognized to be central to the pathogenesis of diabetic kidney disease (Saxena et al., 2019), the understanding of the mechanism of mitochondrial quality control and its regulatory signaling pathways in diabetic kidney disease remains limited.

KCa3.1 (also known as IK1, SK4, or KCNN4) belongs to the calcium-activated potassium channel (KCa) family, which is localized in the plasma membrane, nucleus, and inner mitochondrial membranes (De Marchi et al., 2009; Chachi et al., 2013). KCa3.1 channels regulate calcium entry into cells through modulating calcium-signaling processes, which is necessary for maintaining various cellular activation processes such as proliferation, migration, and cytokine production (Cruse et al., 2006; Wulff and Castle, 2010; Chen et al., 2011). Hence, KCa3.1 has been proposed as a potential therapeutic target for sickle cell anemia, autoimmunity, and atherosclerosis (Wulff et al., 2007; Chou et al., 2008; Wulff and Castle, 2010). Recently, we have demonstrated an important role of KCa3.1 in diabetic kidney disease. Our studies have demonstrated that blockade of KCa3.1 alleviated renal fibrosis and inflammation in diabetic mice through inhibition of the TGF-β1 signaling pathway and fibroblast activation (Huang et al., 2013, 2014b). Furthermore, our results showed that blockade of KCa3.1 is likely to exert its anti-fibrotic effects through the restoration of dysregulated tubular autophagy (Huang et al., 2016a). However, the mechanism by which KCa3.1 mediates mitochondrial quality control in diabetic kidney disease remains unknown.

It is well accepted that transforming growth factor β1 (TGF-β1) plays a central role in the development of diabetic kidney disease. Recent observations indicate an emerging role of TGF-β1 in the regulation of mitochondrial function (Pozdzik et al., 2016; Choi et al., 2019). However, the molecular mechanism mediating mitochondrial quality control in response to TGF-β1 remains limited. In this study, we investigated the effect of KCa3.1 silencing on mitochondrial function in TGF-β1 stimulated human renal proximal tubular cells. We also assessed the role of KCa3.1 in mitochondrial dynamics and mitophagy as well as the underlying signaling pathways in both *in vitro* and *in vivo* models. Our results demonstrated that KCa3.1 deficiency was able to reverse diabetes-induced mitochondrial dysfunction by normalizing the disrupted mitochondrial quality control, which was likely mediated through inhibition of BNIP3 expression.

## Materials and Methods

### Materials

Tissue culture medium and Lipofectamine 2000 were provided from Invitrogen Life Technologies (Carlsbad, CA, United States). Anti-LC3, anti-P62, anti-Cox4, anti-Mfn2, and anti-BNIP3 antibodies were purchased from Abcam (Cambridge, MA, United States), and anti-α-tubulin antibody was from Sigma (St. Louis, MO, United States). Anti-phospho-Drp1 and horseradish peroxidase-conjugated secondary antibodies were purchased from Cell Signaling Technology (Danvers, MA, United States). Anti-Fis1 antibody was purchased from Proteintech (Rosemont, IL, United States), and Anti-Opa1 antibody was purchased from Novus Biologicals (Centennial, CO, United States). Alexa Fluor 488-conjugated secondary antibodies were obtained from Invitrogen (Carlsbad, CA, United States).

### Animal Studies

Male KCa3.1+/+ mice and KCa3.1−/− mice (6–8 weeks old) weighing approximately 20–25 g were used in the study. Mice were intraperitoneally injected with either 55 mg/kg of STZ (Sigma, St. Louis, MO, United States) diluted in 0.1 M citrate buffer, pH 4.5, or citrate buffer alone as described previously (Huang et al., 2013). Mice were weighed, and blood glucose level was determined using the Accu-chek glucometer (Roche Diagnostics). Mice with blood glucose greater than 16 mmol/l were considered to have diabetes.

This study was approved by the Animal Research Ethics Committee of Royal North Shore Hospital (1101-001A). Experimental procedures adhered to the guidelines of the National Health and Medical Research Council of Australia’s Code for the Care and Use of Animals for Scientific Purposes.

### Cell Culture and KCa3.1 Gene Silencing

Immortalized human renal proximal tubular cells (HK2 cells), obtained from ATCC (Manassas, VA, United States), were grown in keratinocyte serum-free media (Invitrogen, Carlsbad, CA, United States). All experiments were performed at passages 5–15.

HK2 cells were transfected with either KCa3.1 siRNA or scrambled control siRNA using Lipofectamine 2000 reagent (Invitrogen, Carlsbad, CA, United States) according to the manufacturer’s instruction. The transfected cells were then incubated with TGF-β1 (2 ng/ml) for 48 h. The siRNA sequence for KCa3.1 is 5′-GCACCUUUCAGACACACUU-3′ (GenePharma, Shanghai).

### Mitochondrial ATP Production Rate

Mitochondrial ATP production rate was determined using the ATP bioluminescence assay kit (Roche Diagnostics, Switzerland) according to the protocol described previously (Huang et al., 2016b). ATP production was induced by incubation of the cell suspension with substrate buffer at 37°C for 10 min, which was then stopped by addition of boiling quenching buffer at 100°C for 2 min. The reaction mixture diluted 1:10 in quenching buffer was measured using an FB10 luminometer (Berthold Detection Systems, Germany) to determine the ATP level.

### Mitochondrial Superoxide Quantification

Mitochondrial superoxide was detected by MitoSOX Red staining (Molecular Probes-Invitrogen) as described previously (Li et al., 2019). Briefly, the treated cells were incubated with 5 μM MitoSOX Red for 15 min at 37°C. After washing with warm buffer, the stained cells were then visualized under confocal fluorescence microscopy (Leica Microsystems, Mannheim, Germany). The results were expressed as the fluorescence intensity normalized to the control group.

### Transmission Electron Microscopy

The cell samples were prepared for transmission electron microscopic analysis as previously reported (Huang et al., 2014a). Briefly, after washing with pre-warmed PBS, the cells were next fixed in 2% glutaraldehyde for 1 h. Subsequently, the fixed cells were postfixed with 1% osmium tetroxide for 1 h after briefly washing with PBS. The samples were rinsed in distilled water, stained with 1% tannic acid, dehydrated in a gradient of ethanol, and embedded in Epon. Sections of 70 nm were generated with an ultramicrotome (Ultracut 7, Leica) and post-stained with 2% aqueous uranyl acetate and Reynold’s lead citrate for 10 min each. The specimens were examined under a transmission electron microscope operating at 200 kV (JEM-2100, JEOL, Japan). Mitochondrial Feret’s diameter (maximum and minimum), the distance between two parallel tangential lines within the selected mitochondrion, was determined using Image J (Demeter-Haludka et al., 2018; Lomash et al., 2019).

### Immunocytofluorescence Staining

To monitor mitophagy, HK2 cells were stained with 1 nM of MitoTracker Deep Red FM for 15 min at 37°C (Huang et al., 2016b). After fixation and blocking, the cells were incubated with primary antibodies against LC3 or P62 in 2% BSA in PBS for 1 h, followed with Alexa Fluor-488 conjugated secondary antibodies for 40 min. The cells were then counterstained and mounted with 4′,6-diamidino-2 phenylindole (DAPI)-mounting medium (Invitrogen). The fluorescent signals were collected and analyzed by confocal fluorescence microscopy (Leica Microsystems, Mannheim, Germany).

### Mitochondrial Isolation

Mitochondria were isolated from mice renal cortex as described previously (Nguyen et al., 2015). Briefly, tissue samples were homogenized in HEPES buffer (20 mM, pH 7.2, containing 1 mM EGTA, 210 mM mannitol, and 70 mM sucrose). The homogenate was centrifuged at 1,500 × *g* for 5 min at 4°C. The supernatant was collected and then centrifuged at 10,000 × *g* for 15 min at 4°C to pellet the mitochondria, which were resuspended in HEPES buffer for analyses. The supernatants were collected as the cytosol fraction. The total protein concentration of the isolated mitochondrial and cytosol fraction was determined by the BCA Protein Assay Kit (Thermo Scientific).

### Western Blotting

An equal amount of cell and tissue lysate samples was separated by SDS-PAGE, and then transferred to Hybond ECL nitrocellulose membrane (Amersham, United States). The membranes were blocked and then probed with primary antibodies (LC3, P62, p-Drp1, Fis1, Opa1, Mfn2, Cox4, BNIP3, and α-Tubulin) at 4°C overnight followed with HRP-conjugated secondary antibody (Amersham, United States). The membrane blots were detected and quantified using LAS-4000 Imaging System (FUJIFILM, Japan).

### Statistical Analysis

The data were expressed as mean ± SEM. Statistical analysis between two groups was evaluated by two-tailed *t*-test. Comparison of the results from multiple groups was performed by one-way ANOVA, followed by Tukey post-test. A *P*-value < 0.05 was considered as statistically significant.

## Results

### KCa3.1 Gene Silencing Reversed TGF-β1-Induced Mitochondrial Dysfunction in HK2 Cells

**FIGURE 1:**
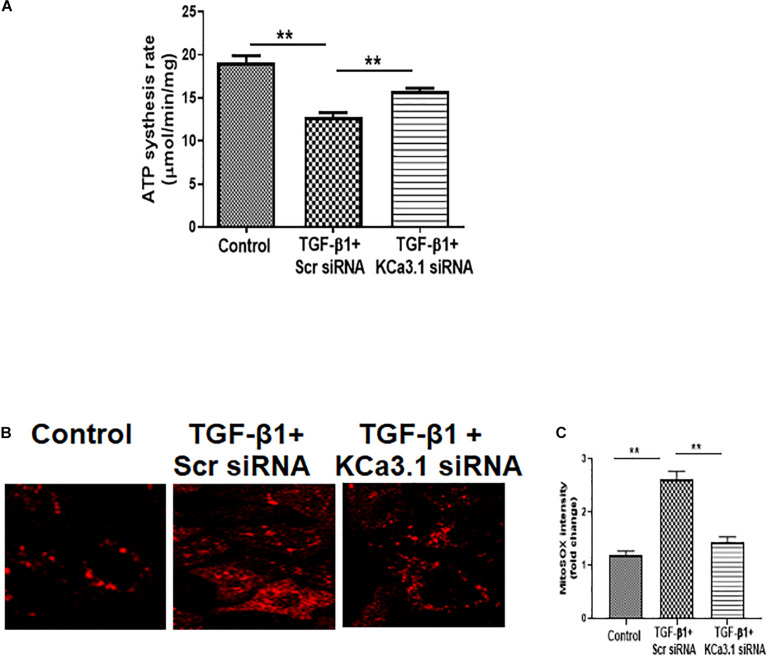
KCa3.1 gene silencing reversed TGF-β1-induced mitochondrial dysfunction in HK2 cells. **(A)** Mitochondrial ATP production rate was assessed to detect mitochondrial function. KCa3.1 silencing significantly increased TGF-β1-induced inhibition of ATP production rate. **(B)** Mitochondrial reactive oxygen species (mtROS) production was assessed by MitoSOX Red staining. KCa3.1 silencing significantly reduced TGF-β1-induced mtROS overproduction. **(C)** Quantification of MitoSOX Red fluorescence intensity normalized to the control group in HK2 cells. Results are presented as mean ± SEM. **P < 0.01. *N* = 3. Original magnification: ×600.

To determine the role of KCa3.1 in mitochondrial function, mitochondrial ATP production rate was first examined in HK2 cells exposed to TGF-β1 with or without KCa3.1 siRNA. As shown in Figure 1A, compared to the controls, TGF-β1 significantly inhibited mitochondrial ATP production rate in HK2 cells transfected with scrambled siRNA (19.08 ± 0.82 for the control and 12.77 ± 0.56 for TGF-β1 + scrambled siRNA, *P* < 0.01, Figure 1A). Inhibition of KCa3.1 with KCa3.1 siRNA reversed TGF-β1-induced inhibition of ATP production rate (15.84 ± 0.29, *P* < 0.01, Figure 1A).

Mitochondrial ROS (mtROS) production in HK2 cells was then examined by fluorescence staining with MitoSOX Red, which is designed for highly selective detection of superoxide in mitochondria. As shown in Figure 1B, a low level of fluorescence was found in the control cells, indicating normal basal levels of mtROS production. Compared to the control cells, TGF-β1 induced increased mtROS production, characterized by the elevated fluorescent intensity of MitoSOX in HK2 cells. KCa3.1 gene silencing significantly reduced TGF-β1-induced mtROS generation (*P* < 0.01, Figure 1C). These data collectively demonstrate that TGF-β1 impaired mitochondrial function through a KCa3.1-related mechanism in HK2 cells and KCa3.1 gene silencing reversed TGF-β1-induced mitochondrial dysfunction.

### KCa3.1 Gene Silencing Attenuated TGF-β1-Induced Increased Fission and Suppressed Fusion in HK2 Cells

**FIGURE 2:**
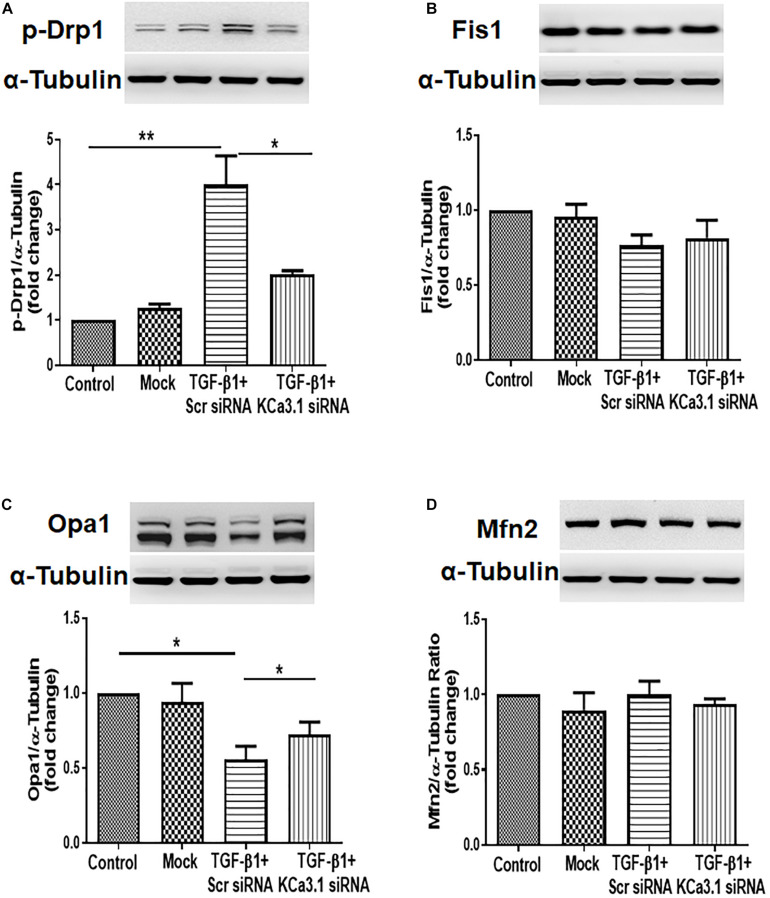
KCa3.1 gene silencing attenuated TGF-β1-induced increased fission and suppressed fusion in HK2 cells. Mitochondrial pro-fission proteins (Drp1 and Fis1) and pro-fusion mediators (Opa1 and Mfn2) were examined by western blotting. Western blot analyses revealed an increased expression of Drp1 **(A)** and a reduced expression of Opa1 **(C)** in TGF-β1-induced HK2 cells, which were reversed by KCa3.1 gene silencing. There were no changes in the expression of Fis1 **(B)** and Mfn2 under TGF-β1 stimulation **(D)**. Results are presented as mean ± SEM. *P < 0.05, **P < 0.01, *N* = 3.

To determine whether KCa3.1 has any effect on mitochondrial fission and fusion processes, mitochondrial fission-related protein Drp1, Fis1, and mitochondrial fusion-related protein Opa1, Mfn2 were examined in HK2 cells exposed to TGF-β1 with or without KCa3.1 gene silencing. As shown in Figure 2A, TGF-β1 significantly increased the level of pro-fission protein Drp1 expression compared to the control group (*P* < 0.01). This increase was attenuated by KCa3.1 gene silencing (*P* < 0.05, Figure 2A). In response to TGF-β1, the levels of profusion protein Opa1 expression in HK2 cells were significantly decreased compared to the control group (*P* < 0.05, Figure 2C), which was attenuated by KCa3.1 gene silencing (*P* < 0.05, Figure 2C). Interestingly, the expression of Fis1 and Mfn2 was not obviously altered by TGF-β1 stimulation (Figures 2B,D).

### KCa3.1 Gene Silencing Reversed TGF-β1-Induced Inhibition of Mitophagy

**FIGURE 3:**
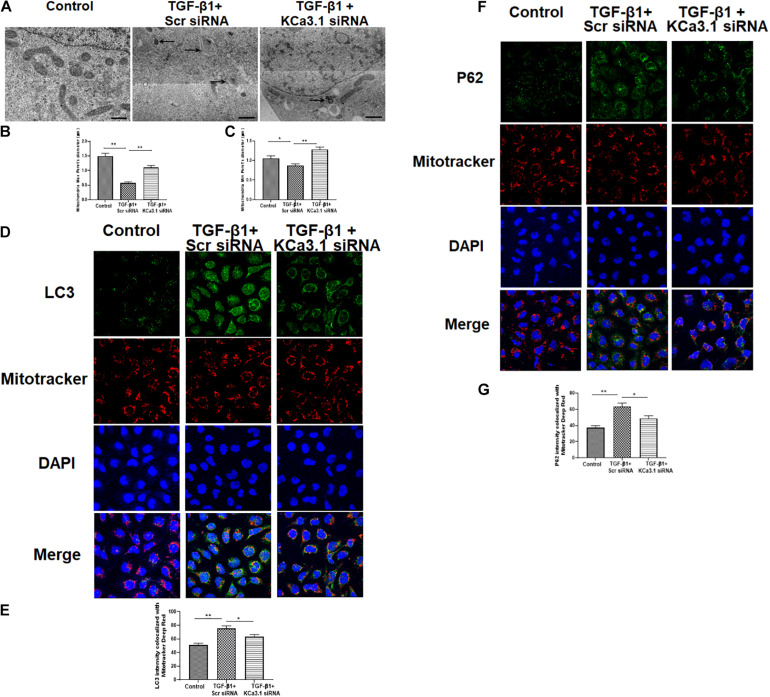
KCa3.1 gene silencing reversed TGF-β1-induced inhibition of mitophagy in HK2 cells. **(A)** Representative electron micrographs of mitochondrial morphology from HK2 cells. The arrow displays abnormalities in mitochondrial morphology indicative of mitophagy in the groups exposed to TGF-β1. Quantification of maximum **(B)** and minimum **(C)** Feret’s diameter in HK2 cells. Scale bars, 200 nm. Confocal microscopy of MitoTracker Red-labeled mitochondria and LC3 staining **(D)** and P62 **(F)**. Quantification of fluorescence intensity of LC3 colocalized with mitochondria **(E)** and P62 colocalized with mitochondria **(G)** in HK2 cells. Results are presented as mean ± SEM. *P < 0.05, **P < 0.01, *N* = 3. Original magnification: ×600.

The mitochondrial shape is maintained through the processes of mitochondrial fission and fusion (Zhang et al., 2019). To investigate the role of KCa3.1 in mitochondrial morphology, we employed transmission electron microscopy to assess the fine structure of mitochondria at high resolution. The control group cells exhibited healthy, normal appearing mitochondria with well-developed cristae (Figure 3A). In contrast, an abundance of mitochondria with severely disrupted cristae was found in HK2 cells exposed to TGF-β1, which was attenuated by KCa3.1 gene silencing. As shown in Figures 3B,C, compared to the control group, exposure to TGF-β1 resulted in a significant reduction in maximum and minimum Feret’s diameter of the mitochondria, indicating that the mitochondria became smaller following the TGF-β1 insult. These alterations were significantly recovered by KCa3.1 gene silencing.

Mitochondrial autophagy was further studied by co-localization of autophagy markers LC3 and P62 with MitoTracker Deep Red stained mitochondria. As shown in Figures 3D,E, the intensity of LC3 that colocalized with MitoTracker Deep Red stained mitochondria was significantly increased in HK2 cells exposed to TGF-β1 when compared to the control (*P* < 0.01, Figure 3E), which was significantly attenuated by KCa3.1 gene silencing (*P* < 0.05, Figures 3D,E). Similarly, exposure of cells to KCa3.1 siRNA significantly suppressed TGF-β1-induced increased intensity of P62 colocalized with the mitochondria (*P* < 0.05, Figures 3F,G). These data indicate that KCa3.1 gene silencing reversed TGF-β1-induced inhibition of mitophagy in HK2 cells.

### KCa3.1 Deficiency Attenuated Diabetes-Induced Increased Fission and Suppressed Fusion in Diabetic Mice

**FIGURE 4:**
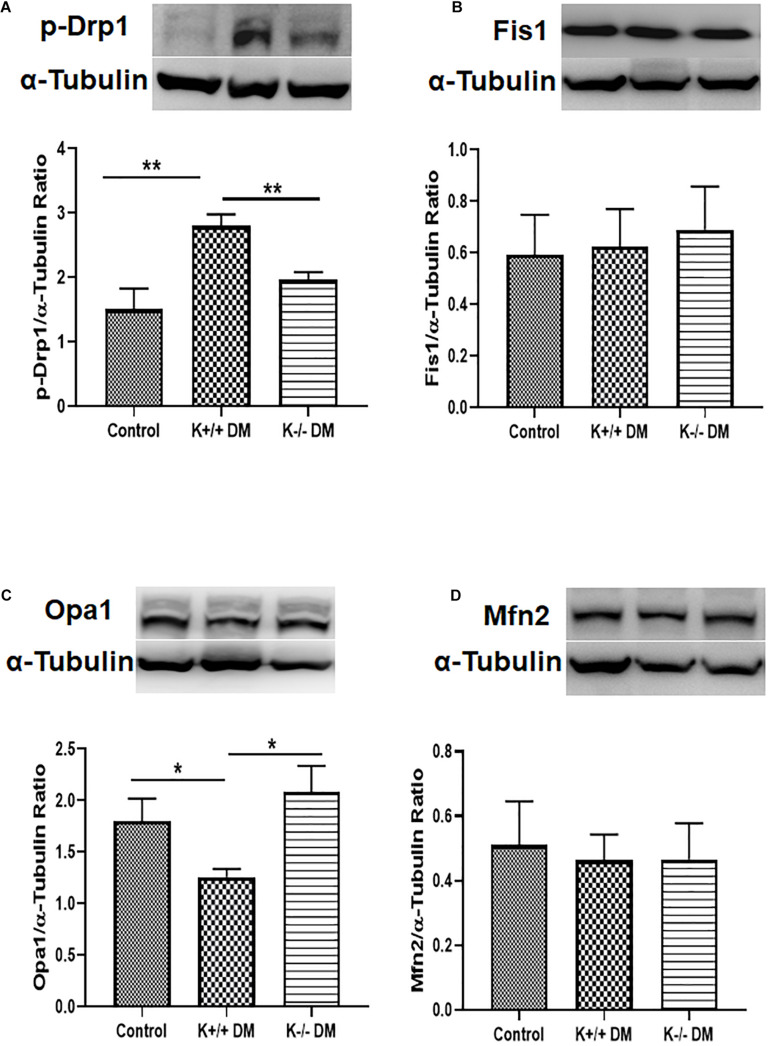
KCa3.1 deficiency attenuated diabetes-induced increased fission and suppressed fusion in diabetic mice. Mitochondrial pro-fission proteins (Drp1 and Fis1) and pro-fusion mediators (Opa1 and Mfn2) were examined by western blotting in kidney tissues. Western blot analysis revealed an increased expression of Drp1 **(A)** and a reduced expression of Opa1 **(C)** in diabetic KCa3.1+/+ mice, which were reversed in KCa3.1 deficient mice (K−/− DM). The levels of Fis1 **(B)** and Mfn2 **(D)** were not notably changed in mice kidneys. Results are presented as mean ± SEM. *P < 0.05, **P < 0.01, *N* = 5.

To further confirm the effect of KCa3.1 on diabetes-related mitochondrial dynamics, mitochondrial fission- and fusion-related proteins were assessed in mice kidneys. As shown in Figure 4, diabetes significantly increased the level of pro-fission protein Drp1 and suppressed the level of pro-fusion protein Opa1 in diabetic KCa3.1 wild-type mice (K+/+ DM) compared to non-diabetic control mice (K+/+ control) (*P* < 0.05, Figures 4A,C). However, the changes were attenuated in diabetic KCa3.1 deficient mice (K−/− DM) (*P* < 0.05, Figures 4A,C). Conversely, the levels of Fis1 and Mfn2 were not notably changed in mice kidneys (Figures 4B,D). The findings suggested that KCa3.1 regulates diabetes-induced imbalance in mitochondrial dynamics by enhancing fission and reducing fusion.

### KCa3.1 Deficiency Attenuated Diabetes-Induced Inhibition of Mitophagy in Diabetic Mice

**FIGURE 5:**
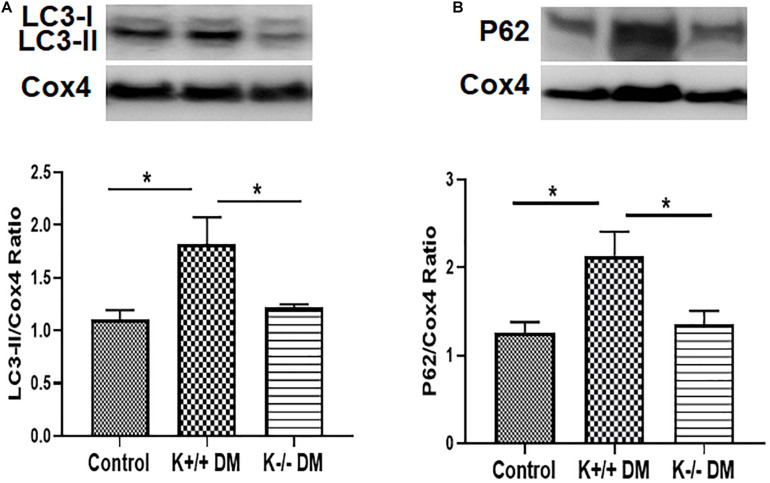
KCa3.1 deficiency attenuated diabetes-induced inhibition of mitophagy in diabetic mice. The autophagy markers LC3 and P62 were assessed in mitochondria from diabetic kidney tissues using western blot analysis. Western blot analyses revealed an increased expression of LC3 **(A)** and P62 **(B)** in diabetic KCa3.1+/+ mice, which were significantly attenuated in KCa3.1 deficient mice (K−/− DM). Results are presented as mean + SEM. *P < 0.05, *N* = 5.

To determine whether KCa3.1 deficiency attenuates diabetic renal fibrosis via regulating mitophagy, the autophagy markers LC3 and P62 were assessed in mitochondria from diabetic kidney tissues using western blot analysis. As shown in Figure 5A, increased expression of LC3 in mitochondria was observed in diabetic KCa3.1 wild-type mice (K+/+ DM) when compared to the non-diabetic controls (K+/+ control) (*P* < 0.05). KCa3.1 deficiency significantly attenuated diabetes-induced upregulation of LC3 expression in mitochondria from diabetic KCa3.1 deficient mice (K−/− DM) (*P* < 0.05, Figure 5A). In line with the LC3 findings, western blot analysis results showed that P62 expression in mitochondria was significantly increased in diabetic kidneys as compared to the non-diabetic controls (*P* < 0.05, Figure 5B), which was inhibited in diabetic KCa3.1 deficient mice (*P* < 0.05, Figure 5B). Collectively, these results indicate that KCa3.1 deficiency attenuates diabetes-induced inhibition of mitophagy in diabetic mice.

### KCa3.1 Deficiency Suppressed Diabetes-Induced Upregulation of BNIP3 Expression in HK2 Cells and Diabetic Mice

**FIGURE 6:**
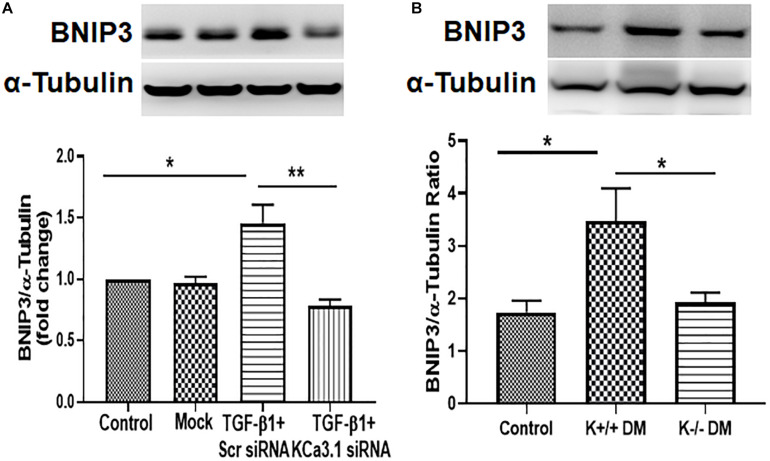
KCa3.1 deficiency suppressed diabetes-induced upregulation of BNIP3 expression in HK2 cells and diabetic mice. **(A)** Western blot analysis showed that KCa3.1 silencing inhibited TGF-β1-induced BNIP3 expression in HK2 cells. *N* = 3. **(B)** Western blot analysis revealed an increased expression of BNIP3 in diabetic KCa3.1+/+ mice, which were significantly attenuated in KCa3.1 deficiency mice (K−/− DM). Results are presented as mean ± SEM. *P < 0.05, **P < 0.01, *N* = 5.

To investigate the mechanism whereby KCa3.1 regulates mitophagy, BNIP3, a regulator of mitophagy, was examined in HK2 cells exposed to TGF-β1 as well as diabetic mice kidneys. As shown in Figure 6A, the expression of BNIP3 was significantly increased by TGF-β1 in HK2 cells (*P* < 0.05, Figure 6A), which was attenuated by KCa3.1 gene silencing (*P* < 0.01, Figure 6A). Similarly, the western blot analysis confirmed a marked induction of BNIP3 in diabetic KCa3.1 wild-type mice (K+/+ DM) when compared to non-diabetic control mice (K+/+ control) (*P* < 0.05, Figure 6B). KCa3.1 deficiency significantly attenuated diabetes-induced upregulation of BNIP3 expression in diabetic KCa3.1−/− mice (K−/− DM) (*P* < 0.05, Figure 6B). Together, these results suggest that KCa3.1-mediated dysregulation of mitophagy is associated with upregulation of BNIP3 expression.

## Discussion

**FIGURE 7:**
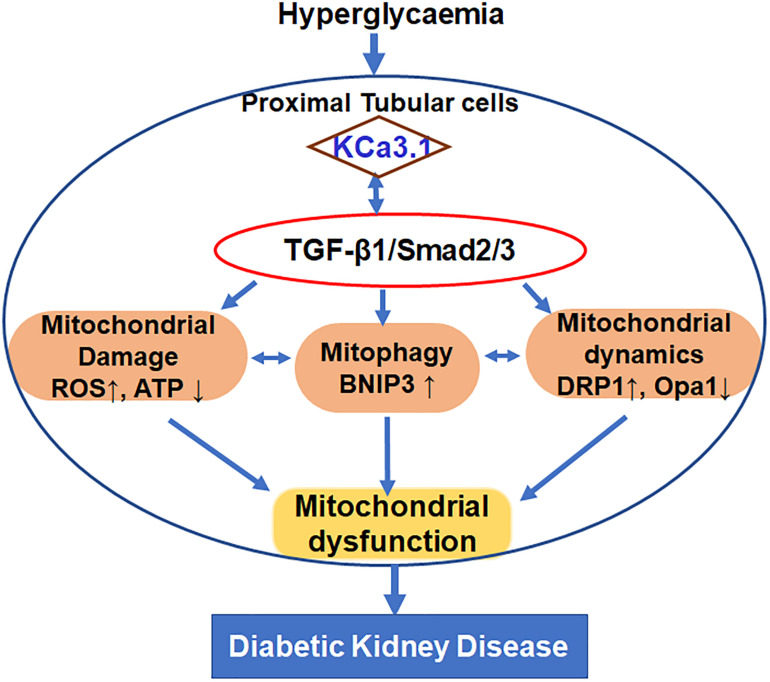
Schematic diagram depicting the conceivable cellular events by which KCa3.1 mediates dysregulation of mitochondrial quality control in diabetic kidney disease.

This study was undertaken to define the role of KCa3.1 in regulating mitochondrial quality control in diabetic kidney disease as depicted in Figure 7. The study demonstrated that TGF-β1 resulted in mitochondrial dysfunction and subsequent mtROS overproduction as well as inhibition of mitophagy, which leads to the disruption of the mitochondrial quality control, eventually causing tubular cell injury. KCa3.1 deficiency restored abnormal mitochondrial dysfunction and mitochondrial quality control by improving BNIP3-mediated mitophagy in TGF-β1-induced renal proximal tubular cells as well as in STZ-induced diabetic mice.

Transforming growth factor β1, the most abundant isoform of TGF-β family members, can be secreted by all types of renal cells and infiltrating inflammatory cells. It is well established that TGF-β1 acts as a pivotal mediator in diabetic kidney disease given its involvement in renal fibrosis, inflammation, cell growth, apoptosis, and differentiation (Bottinger, 2007; Hills and Squires, 2011; Lan, 2012; Meng et al., 2013). A growing body of evidence indicates that mitochondrial dysfunction may be important in the development and progression of diabetic kidney disease (Forbes and Thorburn, 2018; Saxena et al., 2019). Recent studies have revealed a link between TGF-β1 and mitochondrial dysfunction. *In vitro* studies demonstrated that TGF-β1-induced mitochondria dysfunction has been found in various types of cells including lung epithelial cells (Patel et al., 2015), alveolar macrophages (Grunwell et al., 2018), and subepithelial fibroblasts (Sun et al., 2019) as well as renal cells (Yu et al., 2016; Wang Y. et al., 2020). Yu et al. (2016) reported that a TGF-β1-induced fibrotic phenotype was associated with significant mitochondrial dysfunction in mouse renal tubular cells, which was markedly improved by MnTBAP (a cell-permeable mimic of superoxide dismutase) treatment. Recently, mitochondrial dysfunction was found in rat kidney fibroblast cells under TGF-β1 challenge together with fibroblast activation (Wang Y. et al., 2020). Similarly, *in vivo* studies have also demonstrated that increasing TGF-β1 activity is associated with mitochondrial dysfunction and increasing mtROS synthesis in various diseases including diabetic kidney disease (Lee et al., 2017). In line with previous studies, the interaction between TGF-β1 signaling and mitochondria has been demonstrated in the current study. Our previous study showed that the anti-fibrotic effect of KCa3.1 inhibition was likely mediated by antagonizing TGF-β1 signaling through suppression of TGF-β1 and TGF-β receptor II expression and the downstream Smad2/3 pathway in diabetic kidney disease (Huang et al., 2013). Our current results show that TGF-β1 induces mitochondrial dysfunction, as indicated by suppressed ATP production and increased mtROS production in renal proximal tubular cells (Figure 1). Furthermore, our results demonstrate that TGF-β1 exposure leads to altered mitochondrial morphology and increased accumulation of LC3 and P62 colocalized with mitochondria by immunofluorescence staining in renal proximal tubular cells, suggesting that TGF-β1 impaired mitochondrial function and mitophagy flux in renal tubular cells (Figure 3). Taken together, our previous and current studies demonstrate that activation of the TGF-β1 signaling pathway (Huang et al., 2013) and mitochondrial dysfunction are both recovered by KCa3.1 deficiency, indicating that improving mitochondrial function may be a key mechanism by which inhibition of KCa3.1 protects the kidney from diabetes-induced fibrosis.

Mitochondria are dynamic organelles that are constantly undergoing fission and fusion to repair damaged components of the mitochondria and maintain the homeostasis of cells. During mitochondrial fission, Drp1 is recruited from the cytosol onto the MOMs to interact with various receptors, such as Fis1, mitochondrial fission factor (MFF), and mitochondrial dynamic proteins of 49 and 51 kDa (MiD49 and MiD51). Opa1, a dynamin protein, is involved in mitochondrial fusion, cristae structure maintenance, and apoptosis (Del Dotto et al., 2018). Opa1 has eight alternatively spliced isoforms, which can be further processed by proteases yeast mitochondrial escape 1 like 1 ATPase and metalloendopeptidase OMA1 to convert the long Opa1 (L-Opa1) into a cleaved short Opa1 (S-Opa1) form (Anand et al., 2014). L-Opa1 is competent for mitochondrial fusion, while the function of S-Opa1 is still not clear. However, both forms are essential for the function of Opa1 on mitochondrial dynamics and architecture (Del Dotto et al., 2018). The imbalance in mitochondrial fission and fusion largely contributes to tissue pathology in a variety of metabolic conditions, including kidney diseases (Sun et al., 2017; Cassina et al., 2020; Wang Y. et al., 2020; Zhang et al., 2020). Sun et al. (2017) showed expression of the mitochondrial pro-fission protein DRP1 is increased and the mitochondrial pro-fusion protein Opa1 declined in 5/6 nephrectomized (Nx) rats and TGF-β1-exposed HK2 cells. In addition, increased expression of Drp1 and downregulation of Opa1 expression has been found in other animal models of kidney diseases including autosomal dominant polycystic kidney disease, obstructive nephropathy, and STZ-induced diabetic kidney disease (Cassina et al., 2020; Wang Y. et al., 2020; Zhang et al., 2020). Restoration of the imbalanced expression of all these mitochondrial dynamics-associated proteins has been proven to exert renoprotective effects. Consistently, in the present study, excessive mitochondrial fission and decreased fusion have also been demonstrated in TGF-β1-exposed HK2 cells and in kidneys of diabetic mice, as evidenced by upregulation of Drp1 and downregulation of Opa1 (Figures 2, 4). As expected, the long form of Opa1 was downregulated, suggesting decreased fusion. However, the conversion of the long form to the short form of Opa1 was not observed in our study, as indicated by a reduction in the short form of Opa1. The other protease systems such as the ubiquitin proteasome pathway may also be involved in the model of diabetic kidney disease, which deserves further investigation. In our study, KCa3.1 deficiency normalized the expression of mitochondrial dynamic proteins to mitigate the altered mitochondrial dynamics, suggesting that the anti-fibrotic effects of KCa3.1 inhibition may be partly attributed to the modulation of mitochondrial dynamics.

Mitophagy is a form of selective autophagy, which eliminates damaged or defective mitochondria. Recently, mitophagy has emerged as a cytoprotective mechanism to maintain mitochondrial homeostasis and cell survival under conditions of stress. Defective mitophagy has been reported in various kidney diseases including cisplatin-induced acute kidney injury (Zhao et al., 2017), ischemia–reperfusion-induced acute kidney injury (Ishihara et al., 2013), and diabetic kidney disease (Li et al., 2017; Xiao et al., 2017). Consistently, we found that mitophagy was markedly decreased in both *in vitro* and *in vivo* studies (Figures 3, 5), which was accompanied by mitochondrial dysfunction. BNIP3, a member of the Bcl2 family, has been identified as a key receptor for mitophagy via interaction with LC3 (Hanna et al., 2012). BNIP3 resides primarily on the mitochondria and is a critical regulator of mitochondrial function and cell apoptosis (Gao et al., 2020). Specifically, increasing BNIP3 expression leads to loss of mitochondrial membrane potential and the opening of the mitochondrial permeability transformation pore, which results in mitochondrial dysfunction and cell death (Kubli et al., 2007). BNIP3 has been shown to be involved in many diseases such as hepatic, cardiovascular diseases, and cancer (Kanzawa et al., 2005; Dhingra et al., 2017; Gong et al., 2018). In kidneys, Ishihara et al. (2013) observed the induction of BNIP3 together with increased apoptosis and defective autophagy/mitophagy under hypoxic conditions in renal tubular cells and in ischemia–reperfusion injury in rats. Tang et al. (2019) further demonstrated an important role of BNIP3-mediated mitophagy in mitochondrial quality control, tubular cell survival, and renal function during ischemia–reperfusion injury. Recently, Liu et al. (2019) reported that Stanniocalcin-1 ameliorates oxidative stress and cell apoptosis in the kidneys of the db/db mice and high glucose-treated mouse proximal tubular cells by inhibiting BNIP3 expression, which is mediated by activating the AMPK/Sirt3 pathway. In our study, the increased BNIP3 expression was found to be related to dysfunctional mitochondria and abnormal mitochondrial dynamics in TGF-β1-exposed HK2 cells and STZ induced type 1 diabetic mice (Figure 6). KCa3.1 deficiency restored mitochondrial quality surveillance by inhibiting BNIP3 expression, indicating a potential relationship between KCa3.1 and BNIP3. It is important to point out that other mitophagy-related pathways such as PINK1/Parkin and FUNDC1-dependent mitophagy have also been reported in diabetic kidney disease (Xiao et al., 2017; Liu et al., 2020; Wei et al., 2020), which were not examined in this study. Hence, further study is warranted to better understand the role and the interaction between different mitophagy-related pathways in diabetic kidney disease.

## Conclusion

These studies in both *in vitro* and *in vivo* models demonstrate that KCa3.1 mediates dysregulation of mitochondrial function, mitochondrial dynamics, and mitophagy in diabetic kidney disease. Functional KCa3.1 has been shown to be expressed in the inner mitochondrial membrane in addition to the plasma membrane (Leanza et al., 2014; Kovalenko et al., 2016). Although the exact regulatory mechanism of KCa3.1 is not fully understood, it is likely that KCa3.1 regulates mitochondrial quality control through the modulation of membrane potential, cell volume, or calcium influx, which are crucial for mitochondrial function, mitochondrial dynamics, and mitophagy (Mohr et al., 2019; Romero-Garcia and Prado-Garcia, 2019). The findings from the current study not only further confirm the role of mitochondrial dysfunction in diabetic kidney disease, but also offer the potential of targeting KCa3.1 to normalize mitochondrial quality control in the treatment of diabetic kidney disease.

## Data Availability Statement

The raw data supporting the conclusions of this article will be made available by the corresponding author, without undue reservation.

## Ethics Statement

The animal study was reviewed and approved by the Animal Research Ethics Committee of Royal North Shore Hospital.

## Author Contributions

CH, CP, and X-MC conceptualized and designed the experiments. CH, HY, YS, QC, YS, DC, and FB performed the experiments and analyzed the data. CH drafted the manuscript. All authors contributed to manuscript revision and read and approved the final version of the manuscript.

## Conflict of Interest

The authors declare that the research was conducted in the absence of any commercial or financial relationships that could be construed as a potential conflict of interest.
